# Efficacy of growth hormone replacement on anthropometric outcomes, obesity, and lipids in children with optic nerve hypoplasia and growth hormone deficiency

**DOI:** 10.1186/s13633-016-0023-9

**Published:** 2016-03-02

**Authors:** Carly Stewart, Pamela Garcia-Filion, Cassandra Fink, Anna Ryabets-Lienhard, Mitchell E. Geffner, Mark Borchert

**Affiliations:** The Vision Center, Children’s Hospital Los Angeles, 4650 Sunset Boulevard, Los Angeles, CA 90027 USA; Division of Endocrinology, Diabetes, and Metabolism, Children’s Hospital Los Angeles, 4650 Sunset Boulevard, Los Angeles, CA 90027 USA; The Saban Research Institute, Children’s Hospital Los Angeles, 4661 Sunset Boulevard, Los Angeles, CA 90027 USA

**Keywords:** Optic nerve hypoplasia, Growth hormone deficiency, Growth without growth hormone, Obesity

## Abstract

**Background:**

Hypopituitarism and obesity are causes of major lifelong morbidity in patients with optic nerve hypoplasia (ONH). Growth hormone deficiency (GHD) affects the majority of children with ONH, though the degree of deficiency and variability of early growth patterns range from early severe retardation to normal initial growth. The utility of early GH replacement for improving anthropometric, body composition, and lipid outcomes in patients with ONH and GHD, especially those with normal initial height velocity, is unknown. This study examines the effects of GH replacement in a cohort of children with ONH and GHD.

**Methods:**

Controlled clinical trial from 2005–2014. The study included 17 children with ONH and untreated GHD. Those meeting criteria for growth deceleration were assigned to treatment with recombinant human growth hormone (*n* = 5) while those with normal height velocity were randomized either to treatment (*n* = 5) or to observation (no intervention, *n* = 7). Study duration was 3 years. Primary outcome measures included stature, weight, weight-for-stature, and BMI standard deviation score (SDS) at study completion.

**Results:**

Subjects on GH, irrespective of entry growth trajectory, grew more on average in stature than controls by a difference of 0.98 SDS by study end; this effect persisted after adjusting for baseline overweight status. Treatment had an effect on weight SDS only after adjusting for initial overweight status, resulting in an average increase of 0.83 SDS more than controls. Subjects who were overweight at the outset experienced greater gains in both weight and stature SDS. Treatment had no statistically significant impact on weight-for-stature or BMI SDS. A reduction in body fat percentage was observed in those treated, both before (−6.1 %) and after (−4.3 %) adjustment for initial overweight status.

**Conclusion:**

Early GH replacement has a positive effect on short-term statural outcomes in children with ONH and GHD, even in those exhibiting normal initial linear growth. Results were less conclusive regarding treatment effects on body composition and lipids.

## Background

Optic nerve hypoplasia (ONH) is associated with endocrine and metabolic manifestations, such as hypothalamic dysregulation, hypopituitarism, and/or obesity [1–3]. Growth hormone deficiency (GHD) is the predominant endocrinopathy, affecting as many as 70 % of children with ONH. Classically, GHD presents as slow linear growth, but normal growth velocity despite GHD is a documented phenomenon among children with ONH. Suppositions for this paradoxical “growth without growth hormone (GH)” include the presence of alternative growth-promoting factors and obesity itself, which may affect as many as 50 % of patients with ONH [1, 4, 5]. It has been reported that some ONH patients with GHD and seemingly normal initial height velocity eventually experience deceleration in growth, around age 3.5 years [6]. What is unknown is whether early treatment with GH in these patients is effective in improving height outcomes or, secondarily, in preventing or mitigating obesity or hyperlipidemia, benefits reported to occur in adults with exogenous obesity and GHD after GH replacement [1, 5, 7, 8].

The Vision Center at Children’s Hospital Los Angeles is a referral center for diagnosis and management of children with ONH. Using the Center’s prospective registry of patients with ONH, we conducted a controlled clinical trial to examine the effect of early GH replacement therapy on anthropometric measurements, body composition, and lipid profiles in children with ONH and GHD.

## Methods

### Study design

Subjects were recruited from a prospective clinical registry of young children with ONH. Detailed information about the registry cohort has been previously described [1, 9]. As part of the registry, all patients undergo a one-time GH provocation test by glucagon stimulation prior to 5 years of age regardless of growth status. Prior to September 2009, the methodology for measuring GH used radioimmunoassay (Endocrine Sciences), which matched the WHO international reference preparation 66/217 recombinant standard; subsequent tests were measured by immunoassay (Siemens Immulite 2000) with calibration equaling WHO international standard 98/574. GHD was defined as a subnormal peak GH response (<10 ng/mL). Patients with GHD and no previous GH therapy met study inclusion criteria. At enrollment, subjects underwent a baseline endocrinological evaluation prior to being assigned to a treatment group (GH replacement *versus* observational control) using the criteria described below. The study follow-up duration was 3 years. Research was in compliance with the Helsinki Declaration and approved by the Institutional Review Board at Children’s Hospital Los Angeles; written informed consent was obtained from participants’ parents or guardians.

### Study measurements

Following enrollment (baseline), subjects were seen every 4 months for sequential anthropometric measurements and laboratory testing. The former included stature (length for subjects < 36 months; height for those ≥ 36 months), weight, weight-for-stature (if < 121.5 cm), and body mass index (BMI) (after age 2 years) for all subjects. To calculate BMI for subjects aged 24–36 months, recumbent lengths were converted to heights by subtracting 0.8 cm [10]. Absolute measurements were normalized for age and sex by conversion to z-scores (standard deviation score; SDS) using Epi Info Version 3.5.4 (Centers for Disease Control and Prevention, Atlanta, GA). Length SDS and height SDS were combined for analyses of stature SDS. Weight-for-stature SDS data were derived from weight-for-length for subjects < 36 months and weight-for-height for those ≥ 36 months. Growth velocity (cm/year) is given as median and interquartile range; it was not converted to SDS due to the age range of the cohort, as no single reference provides growth velocity percentiles for children both under and over age 2 years. Body composition measurements of body fat percentage were estimated using bioelectrical impedance (Biodynamics, Seattle, WA).

Pre-enrollment laboratory testing of GH surrogates (IGF-I and IGFBP-3), thyroid function (free T4 by dialysis), and a fasting lipid panel (total, LDL, and HDL cholesterol, and triglycerides) was performed on all subjects, and at each study visit for subjects in the GH replacement group. Subsequent laboratory testing for subjects in the control group included a fasting lipid panel at the initial and final visits only. Additional study data, obtained from subjects’ medical records, included length and height measurements over the year preceding enrollment, serum prolactin levels, the presence of other endocrinopathies, and abnormalities identified on neuroradiographic imaging.

### Groups

Treatment group assignment was based on subjects’ stature SDS relative to the predicted mid-parental target height (MPTH) at baseline and subsequent classification as having growth deceleration or normal growth. MPTH was calculated as the average of the parents’ heights, less 2.5 inches for girls, plus 2.5 inches for boys. Criteria for growth deceleration were either stature at least 2 SDS below the MPTH (or the 50^th^ percentile for height for age in the absence of MPTH, as was the case for one adopted subject) along with a 1 SDS drop in length/height, or a stature at least 3 SDS below the MPTH along with any decrease in length/height SDS. Subjects with growth deceleration so defined were assigned to the GH replacement treatment group. Subjects who did not meet these criteria were classified as having normal growth and were randomly assigned to GH replacement or to an observational control group (no GH replacement) [11]. Data from the two GH-treated groups were combined to assess the efficacy of this treatment approach irrespective of entry growth velocity. For the untreated control group, subjects were monitored for change in growth trajectory. Control subjects were switched (crossed over) to the GH replacement group if, during the course of the study, they met criteria for growth deceleration as previously defined. The study drug was Nutropin AQ® (Genentech, South San Francisco, CA). The starting dose for GH replacement was calculated as 0.3 mg/kg/week and subsequently modified by a study endocrinologist based on observed length/height velocity and serum IGF-I levels. GH surrogates were also used to monitor compliance with treatment.

### Data analysis

The primary outcome measures were stature, weight, weight-for-stature, and BMI SDS. The study hypotheses were that, by the end of the three-year trial, the mean SDS for stature would be higher and weight, weight-for-stature, and BMI SDS would be lower for subjects in the GH replacement group compared to the control group.

Linear regression models were constructed to analyze the outcomes as a function of the original treatment group assignments (GH replacement *versus* control) regardless of treatment status at completion. The GH replacement group consisted of subjects both assigned and randomized to GH; collapsing was deemed appropriate after sensitivity analysis demonstrated similar results in each of the two groups separately. The linear regression analysis used the generalized estimating equation method to determine the average effect of GH replacement therapy on stature, weight, weight-for-stature, and BMI SDS with model-based variance estimation [12]. To account for the within-subject dependency of repeated measurement, an exchangeable working correlation matrix was specified [13]. Presence of growth deceleration (described in the prior section) at baseline or any point during follow-up was included as a covariate to control for potential treatment bias, allowing cross-over subjects to be analyzed by their initial group assignment, in an intent-to-treat manner. Regression coefficients [increase or decrease (−)], interpreted as the average response to treatment compared to no treatment (control), are presented with standard error (± SE) and 95 % confidence interval (95 % CI).

Body composition and lipid levels were secondary outcomes of the efficacy of GH replacement therapy. For body composition, analysis assessed whether body fat percentage based on bioelectrical impedance decreased in response to GH replacement. Analysis of body composition used the generalized estimating equation method as previously described. Lipid levels were examined for categorical (normal *versus* abnormal) changes: increase in HDL and decrease in total and LDL cholesterol, and triglycerides.

To examine the influence of baseline overweight status on efficacy of GH replacement, the regression estimates were adjusted for subject status as overweight, underweight, or normal weight. In accordance with the 2000 Centers for Disease Control growth charts, overweight was defined as: weight-for-stature above the 95^th^ percentile for subjects under 24 months of age or a BMI greater than the 85^th^ percentile for subjects 24 months and older [14]; and underweight as weight-for-stature or BMI below the 5^th^ percentile.

Analyses were performed with Stata 11.0 SE (College Station, Texas). Study data are presented using descriptive statistics; proportions for categorical data and median (interquartile range; IQR) for continuous data. Regression estimates are presented as unadjusted and adjusted (for baseline weight status).

## Results

### Study participants

**Fig. 1:**
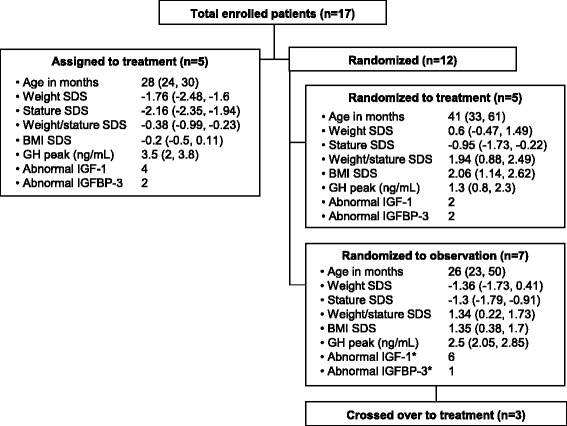
Assignment groups and initial characteristics at enrollment (presented as medians and interquartile ranges; IGF-I and IGFBP-3 are presented as absolute number of subjects). ^*^ Results unavailable for one subject

The study included 17 participants (male = 8) enrolled at a median age of 30 months (IQR: 24, 50). The serum GH level after stimulation peaked at a median value of 2.3 ng/mL (IQR: 1.4, 3.5). Table 1 presents the clinical profile of the cohort. Treatment group assignments are illustrated in Fig. 1. Pre-enrollment IGF-I and IGFBP-3 status are presented in Fig. 1, though these were not considered methodologically in our definition and diagnosis of GHD. Baseline growth velocity in the group assigned to GH replacement was 5.28 cm/year, 7.2 cm/year in those with normal baseline growth velocity who were randomized to GH replacement, and 7.4 cm/year in untreated controls.

**Table 1:** Clinical profile of the study cohort (*n* = 17)

	Number	Percent
Unilateral ONH	1	6
Overweight at baseline	7	41
Underweight at baseline	2	11
Brain malformations		
Hypoplastic corpus callosum	10	60
Absent septum pellucidum	7	41
Major cerebral malformation	3	18
Pituitary gland malformation	0	0
Non-visualized neurohypophysis^a^	3	18
Hypopituitarism (in addition to GH)		
Hypothyroidism	8	47
Adrenal insufficiency	6	35
Diabetes insipidus	3	18

^a^All with infundibulum visualized

Three subjects initially assigned to the control group crossed over to the GH replacement group at follow-up visits 3 (2 subjects) and at follow-up visit 6 (1 subject) due to growth deceleration. All subjects underwent routine laboratory tests for hypothyroidism, and any observed deficiency was treated per standard of care.

There were 8 subjects (6 in GH replacement group) with normal weight status at baseline, and all remained so at the final study visit. Seven children were classified as overweight at baseline; 3 normalized (2 on GH replacement) and 4 remained overweight (3 on GH) at the final visit. Two subjects were underweight at baseline; 1 remained underweight and 1 was overweight at the final visit (both crossed over to GH replacement).

### Anthropometric outcomes

**Fig. 2:**
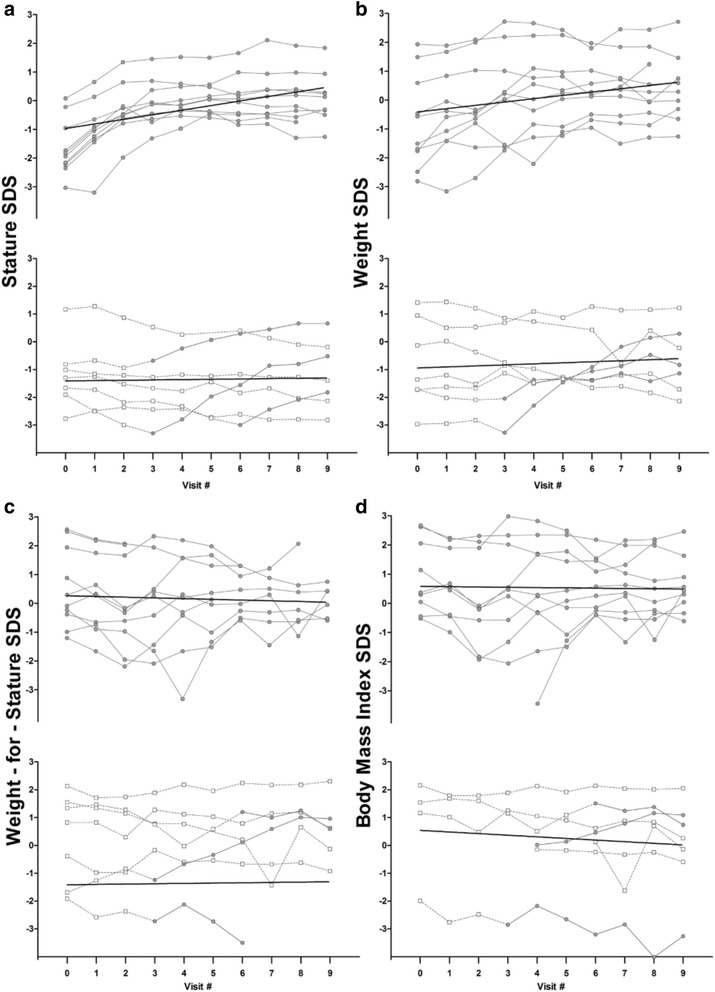
Anthropometric SDS measurements from baseline (visit 0) through follow-up (visit 9) for **a**) stature, **b**) weight, **c**) weight-for-stature, and **d**) BMI. Treatment groups: solid line () for GH replacement and dashed line () for control. For subjects that started as controls, but crossed over to treatment during follow-up, lines change from dotted to solid at the point of cross-over

The treatment effects of GH replacement on outcomes are presented in Table 2. Subjects in the GH replacement group experienced a greater average increase in stature (0.98 SDS; 95 % CI: 0.14, 1.82) than control subjects (p_unadj_ = 0.022). The effect of GH replacement on stature was unaffected by baseline average overweight status (0.96; 95 % CI: 0.09, 1.82, p_adj_ = 0.030). An increase in average weight SDS was associated with GH replacement (0.83 SDS; 95 % CI: 0.04, 1.62) after adjusting for baseline overweight status (p_adj_ = 0.04, p_unadj_ = 0.251). Subjects who were overweight at baseline experienced greater gains in both weight and stature. GH replacement did not affect weight-for-stature (p_unadj_ = 0.808) or BMI (p_unadj_ = 0.959) SDS, an outcome which persisted even after adjusting for baseline overweight status. The primary outcome measurements by treatment group are illustrated in Fig. 2. Median growth velocity in control subjects slowed to a mean of 5.7 cm/year (IQR: 4.8, 5.9) by the final study visit (or prior to cross-over, in applicable cases). Growth velocity at study completion for treated subjects was 9.5 cm/year (IQR: 7.8, 9.8). Subdivided by group assignment, growth velocity was 9.7 cm/year (IQR: 9.3, 10.0) in the group assigned to GH replacement and 8.4 cm/year (IQR: 7.0, 9.6) in those randomized to GH.

**Table 2:** Treatment effect of GH on anthropometric and body composition outcomes

	Unadjusted			Adjusted^a^		
	β^b^ ± SE	95 % CI	*p-value*	β^b^ ± SE	95 % CI	*p-value*
Stature SDS	0.98 ± 0.43	0.14, 1.82	0.022	0.96 ± 0.44	0.09, 1.82	0.030
Weight SDS	0.55 ± 0.48	−0.39, 1.50	0.251	0.83 ± 0.40	0.04, 1.62	0.040
BMI SDS	−0.03 ± 0.57	−1.15, 1.09	0.959	0.40 ± 0.43	−0.44, 1.25	0.349
Weight-for-Stature SDS	−0.12 ± 0.50	−1.11, 0.87	0.808	0.26 ± 0.35	−0.44, 0.95	0.468
Body Fat (%)	−6.1 ± 2.8	−11.5, −0.6	0.030	−4.3 ± 2.3	−8.8, 0.1	0.057

^a^Adjusted for overweight status at baseline (normal/underweight/overweight)

^b^β represents regression coefficient, interpreted as the average response [increase or decrease (−)] to GH replacement compared to no replacement

### Body composition and lipid level outcomes

Subjects’ body fat percentage showed a modest decline regardless of adjustment for baseline overweight status (Table 2). Prior to adjustment, GH replacement effected a mean 6.1 % (95 % CI: −11.5 %, −0.6 %) decrease in body fat (p_unadj_ = 0.030); after adjustment for weight, the mean decrease was 4.3 % (p_adj_ = 0.057). Total cholesterol levels changed from abnormal (high or low) at baseline to normal at study completion in 4 subjects (3 on GH replacement); LDL normalized in 4 (all on GH), and HDL changed from low to normal in 5 (all on GH). Total cholesterol changed from normal to high in 3 (2 on GH) while HDL and triglycerides each changed from normal to low in 1 subject (on GH).

## Discussion

This study was designed to examine the benefit of early GH replacement therapy on growth, body composition, and serum lipids in young children with ONH and GHD, and to explore whether there is any indication for early GH treatment even in those with normal initial growth. The results suggest that nearly all children with ONH and GHD ultimately warrant GH replacement for growth reasons. In this study, 6 of the 7 control subjects were eventually assigned to treatment for growth deceleration. Three were assigned during the course of this study, at a median age of 51 months, while 3 of the remaining controls were assigned upon study completion at a median age of 60 months. This finding is consistent with previous reports of eventual growth deceleration in ONH patients with GHD that occurred around age 3.5 years, despite normal initial growth velocity [6].

Whether or not early GH replacement is indicated in patients with normal growth depends on the efficacy of the drug to improve their overall growth in height and/or mitigate the problem of excess weight and its co-morbidities. To the first end, our findings demonstrate a positive effect of GH replacement on stature; in fact, children randomized to GH replacement while their growth was normal exhibited a greater response to treatment than those assigned to GH due to growth deceleration. The effect on body composition, however, was less conclusive.

Researchers have proposed that GH replacement may mitigate the development of excessive weight in children with ONH and GHD [1, 15]. Studies of GH replacement in obese, otherwise healthy adults have yielded various benefits, including the reduction of total body fat and abdominal adipose tissue; lowering of total cholesterol, LDL, and triglycerides; increasing of HDL; and promotion of overall weight loss [7, 8, 16]. Additional studies have shown a favorable impact of GH replacement on body composition and lipid metabolism in children with isolated GHD [17, 18]. Further, GH replacement has been shown to reduce body fat percentage and increase lean body mass in patients with Prader-Willi syndrome (PWS) and Turner syndrome who, like those with ONH, typically manifest short stature and obesity [19, 20].

Subjects who were overweight at the outset of this study experienced greater gains in both weight and stature with no accompanying decrease in BMI or weight-for-stature. On the other hand, GH replacement was associated with a decrease in body fat percentage of approximately 4-6 %, a finding similar to the improvement in body fat percentage observed in a 12-month randomized controlled study of GH replacement in PWS patients, as measured by dual-energy x-ray absorptiometry (DEXA) [19]. Since BMI is not validated for our youngest study participants, nor weight-for-height for the tallest, exclusion of these subjects’ respective data may have impacted the detection of a treatment effect. Observed changes in serum lipids were mostly positive; while all subjects with abnormal total, LDL, and/or HDL cholesterol at baseline, who were treated with GH, attained normal levels by study completion, 2 subjects on GH saw an unfavorable change in total cholesterol, rendering the overall treatment effect to be inconclusive.

On the whole, these results suggest that obesity in children with ONH is not solely attributable to GHD and cannot be resolved simply by correcting that deficiency. Overweight subjects’ stature responded well to GH – better, in fact, than their normal-weight peers – but they did not experience a decrease in BMI or weight-for-stature SDS. Importantly, those subjects with normal initial linear growth were markedly more overweight (7/12) than those assigned to GH treatment (0). This points to the central question of what causes excess weight in ONH, and to the related question of what drives growth in spite of GHD. Obesity in this population is likely linked to hypothalamic dysfunction, and a result of hyperphagia, hyperleptinemia or reduced leptin sensitivity, and/or low sympathetic or dopaminergic tone [5, 21]. Obesity itself is associated with suppressed GH secretion, as well as with hyperleptinemia [1, 22]. Accordingly, stimulated GH peaks in this study were lower among overweight subjects compared to their non-overweight peers. This could rightfully raise doubts as to the true deficiency status of overweight individuals; however, the fact that almost all of our control subjects experienced eventual growth deceleration does substantiate their GHD.

Another proposed driver of growth without GH, hyperprolactinemia, which may be a marker of hypothalamic dysfunction and was found in 88 % of our subjects, has been shown to correlate with the presence of obesity in normal children and adults. However, an earlier study of patients with ONH by Vedin et al. detected no association between elevated prolactin and BMI by 5 years of age [5, 15, 23]. Prolactin itself has been suggested to increase serum somatomedin C/IGF-I levels in patients with craniopharyngioma or pituitary tumors who exhibit normal IGF-I levels and normal-to-accelerated growth following surgery; this possibility is based on *in vitro* mitogenic properties of prolactin upon mammary tissue proliferation in rodents and cell division in Nb2 node lymphoma cells [4, 24, 25]. However, this mechanism cannot fully explain growth without GH in ONH patients because serum prolactin levels are not universally high and, when elevated, are only mildly so. Furthermore, despite hyperprolactinemia in some cases of ONH, circulating IGF-I levels are frequently decreased [6, 24, 26]. Insulin, which drives growth in the prenatal period, has been cited as a possible mechanism behind the “growth without GH” syndrome, as obesity-induced hyperinsulinemia may stimulate hepatic production of IGF-I. However, abnormally high levels of insulin occur in only a subset of children with ONH, and previous authors have ruled out hyperinsulinemia as a growth factor in their respective studies of this patient population [4, 6, 24, 26]. Finally, the presence of an as yet uncharacterized circulating growth factor cannot be ruled out [27].

The predominant limitation of this study was its small sample size. Moreover, the unavailability of BMI for children < 2 years and of weight-for-stature for children > 121.5 cm in height, imposed further constraints on the analysis. Notwithstanding, the statistical significance achieved with such a small sample suggests that the effects are real. BMI itself is an imperfect indicator of obesity, though it is the preferred measure in children [14]. Furthermore, while bioelectrical impedance is a practical method to assess body fat in children and has been validated against other models (densitometry and DEXA) [28], there is significant variability in devices and prediction algorithms, and results may be influenced by fasting, hydration, body position, and other variables [29]. Other methods of measurement, including waist circumference, waist-to-hip or waist-to-height ratios, skinfold thickness, and DEXA, may provide further reliability in assessing body composition [14]. Lastly, GHD status and eligibility were determined from only one stimulation test (glucagon), which may seem a narrow means of diagnosis. However, according to consensus guidelines issued by the Growth Hormone Research Society, which stipulate a second stimulation be used in cases of isolated GHD, only one test – in combination with clinical, auxological, and radiological assessment – suffices for patients with defined central nervous system pathology and/or multiple pituitary hormone deficiencies [30].

## Conclusions

Eventually, nearly all patients with ONH and GHD will require GH replacement for growth deceleration. While early treatment appears to have a profound effect on short-term height trajectory, even in children who are growing normally, the results of this trial are less clear with regard to acute effects on body composition. Despite an observed reduction in body fat percentage, GH replacement did not appear to improve weight-for-stature or BMI in treated subjects. This research constitutes a first attempt to study the efficacy of GH to mitigate excess weight in ONH. Its findings underscore the suspicion that obesity is attributed to hypothalamic dysfunction and cannot be remedied with GH replacement alone.

## Abbreviations

BMI: Body mass index
GH: Growth hormone
GHD: Growth hormone deficiency
ONH: Optic nerve hypoplasia
SDS: Standard deviation score

## References

[1] Ahmad T, Garcia-Filion P, Borchert M, Kaufman F, Burkett L, Geffner M (2006). Endocrinological and auxological abnormalities in young children with optic nerve hypoplasia: a prospective study. J Pediatr.

[2] Ma NS, Fink C, Geffner ME, Borchert M (2010). Evolving central hypothyroidism in children with optic nerve hypoplasia. J Pediatr Endocrinol Metab.

[3] Haddad NG, Eugster EA (2005). Hypopituitarism and neurodevelopmental abnormalities in relation to central nervous system structural defects in children with optic nerve hypoplasia. J Pediatr Endocrinol Metab.

[4] Geffner ME, Lippe BM, Bersch N, Van Herle A, Kaplan SA, Elders MJ (1986). Growth without growth hormone: evidence for a potent circulating human growth factor. Lancet.

[5] Vedin AM, Garcia-Filion P, Fink C, Borchert M, Geffner ME (2012). Serum prolactin concentrations in relation to hypopituitarism and obesity in children with optic nerve hypoplasia. Horm Res Paediatr.

[6] Costin G, Murphree AL (1985). Hypothalamic-pituitary function in children with optic nerve hypoplasia. Am J Dis Child.

[7] Albert SG, Mooradian AD (2004). Low-dose recombinant human growth hormone as adjuvant therapy to lifestyle modifications in the management of obesity. J Clin Endocrinol Metab.

[8] Johannsson G, Marin P, Lonn L, Ottosson M, Stenlof K, Bjorntorp P (1997). Growth hormone treatment of abdominally obese men reduces abdominal fat mass, improves glucose and lipoprotein metabolism, and reduces diastolic blood pressure. J Clin Endocrinol Metab.

[9] Garcia-Filion P, Epport K, Nelson M, Azen C, Geffner ME, Fink C (2008). Neuroradiographic, endocrinologic, and ophthalmic correlates of adverse developmental outcomes in children with optic nerve hypoplasia: a prospective study. Pediatrics.

[10] Kuczmarski RJ, Ogden CL, Guo SS, Grummer-Strawn LM, Flegal KM, Mei Z (2002). 2000 CDC Growth Charts for the United States: methods and development. Vital Health Stat.

[11] Berry DA, Eick SG (1995). Adaptive assignment versus balanced randomization in clinical trials: a decision analysis. Stat Med.

[12] Zeger SL, Liang KY (1986). Longitudinal data analysis for discrete and continuous outcomes. Biometrics.

[13] Wang Y, Carey V (2003). Working Correlation Structure Misspecification, Estimation and Covariate Design: Implications for Generalised Estimating Equations Performance. Biometrika.

[14] Krebs NF, Himes JH, Jacobson D, Nicklas TA, Guilday P, Styne D (2007). Assessment of child and adolescent overweight and obesity. Pediatrics.

[15] Vedin AM, Karlsson H, Fink C, Borchert M, Geffner ME (2011). Presenting features and long-term effects of growth hormone treatment of children with optic nerve hypoplasia/septo-optic dysplasia. Int J Pediatr Endocrinol.

[16] Claessen KM, Appelman-Dijkstra NM, Adoptie DM, Roelfsema F, Smit JW, Biermasz NR (2013). Metabolic profile in growth hormone-deficient (GHD) adults after long-term recombinant human growth hormone (rhGH) therapy. J Clin Endocrinol Metab.

[17] De Marco S, Marcovecchio ML, Caniglia D, De Leonibus C, Chiarelli F, Mohn A. Circulating asymmetric dimethylarginine and lipid profile in pre-pubertal children with growth hormone deficiency: Effect of 12-month growth hormone replacement therapy. Growth Horm IGF Res. 2014. 10.1016/j.ghir.2014.08.001 25172154

[18] Sas T, Mulder P, Hokken-Koelega A (2000). Body composition, blood pressure, and lipid metabolism before and during long-term growth hormone (GH) treatment in children with short stature born small for gestational age either with or without GH deficiency. J Clin Endocrinol Metab.

[19] Carrel AL, Moerchen V, Myers SE, Bekx MT, Whitman BY, Allen DB (2004). Growth hormone improves mobility and body composition in infants and toddlers with Prader-Willi syndrome. J Pediatr.

[20] Hjerrild BE, Mortensen KH, Gravholt CH (2008). Turner syndrome and clinical treatment. Br Med Bull.

[21] Roth CL, Hunneman DH, Gebhardt U, Stoffel-Wagner B, Reinehr T, Muller HL (2007). Reduced sympathetic metabolites in urine of obese patients with craniopharyngioma. Pediatr Res.

[22] Carro E, Senaris R, Considine RV, Casanueva FF, Dieguez C (1997). Regulation of in vivo growth hormone secretion by leptin. Endocrinology.

[23] Doknic M, Pekic S, Zarkovic M, Medic-Stojanoska M, Dieguez C, Casanueva F (2002). Dopaminergic tone and obesity: an insight from prolactinomas treated with bromocriptine. Eur J Endocrinol.

[24] Bereket A, Lang CH, Geffner ME, Wilson TA (1998). Normal growth in a patient with septo-optic dysplasia despite both growth hormone and IGF-I deficiency. J Pediatr Endocrinol Metab.

[25] Clemmons DR, Underwood LE, Ridgway EC, Kliman B, Van Wyk JJ (1981). Hyperprolactinemia is associated with increased immunoreactive somatomedin C in hypopituitarism. J Clin Endocrinol Metab.

[26] Hathout EH, Baylink DJ, Mohan S (1999). Normal growth despite GH, IGF-I and IGF-II deficiency. Growth Horm IGF Res.

[27] Geffner ME (1996). The growth without growth hormone syndrome. Endocrinol Metab Clin North Am.

[28] Mast M, Sonnichsen A, Langnase K, Labitzke K, Bruse U, Preub U (2002). Inconsistencies in bioelectrical impedance and anthropometric measurements of fat mass in a field study of prepubertal children. Br J Nutr.

[29] Talma H, Chinapaw MJ, Bakker B, HiraSing RA, Terwee CB, Altenburg TM (2013). Bioelectrical impedance analysis to estimate body composition in children and adolescents: a systematic review and evidence appraisal of validity, responsiveness, reliability and measurement error. Obes Rev.

[30] Growth Hormone Research S (2000). Consensus guidelines for the diagnosis and treatment of growth hormone (GH) deficiency in childhood and adolescence: summary statement of the GH Research Society. GH Research Society. J Clin Endocrinol Metab.

